# Disparities in breast cancer incidence and survival by age, race, and molecular subtype in US women

**DOI:** 10.1038/s41523-026-00935-y

**Published:** 2026-03-27

**Authors:** Lin Wang, Zhihao Wan, Vikramjit Dhillon, Xin Wang, Zheng Yin, Chika F. Ezeana, Polly Niravath, Akshjot Puri, Kai Sun, Jenny Chang, Stephen T. C. Wong

**Affiliations:** 1 Department of System Medicine and Bioengineering, Houston Methodist Neal Cancer Center, Houston Methodist Hospital, Houston, TX USA; 2 Houston Methodist Neal Cancer Center, Houston Methodist Hospital, Houston, TX USA; 3 Houston Methodist Academic Institute, Houston Methodist Hospital, Houston, TX USA

**Keywords:** Cancer, Oncology, Risk factors

## Abstract

Using SEER datasets spanning nearly five decades, we identified a striking longitudinal shift in the breast cancer mortality burden from older to younger women, revealing an evolving risk landscape that warrant challenges to prevailing assumptions about age-related vulnerability. Leveraging contemporary data (2010–2022), we examined how molecular subtype, race, and age intersect jointly to shape survival disparities. As expected, young Black women with triple-negative breast cancer (HR−/HER2−) exhibited significantly elevated hazard ratios, underscoring their urgent need for targeted interventions and prevention strategies. Importantly, we also uncovered disproportionately high mortality risks among Asian women: those under 50 years experienced poorer outcomes for triple-negative breast cancer. These findings suggest that distinct biological, hormonal, and sociodemographic factors may contribute to heterogeneity in cancer progression and survival across populations. In addition, demographic analyses revealed that Asian and Hispanic women have steadily increased in proportional representation, surpassing Black patients recently, underscoring critical population shifts in the breast cancer burden. Together, these results not only broaden current understanding of how age, ancestry, and molecular subtype converge to drive disparities, but also underscore the urgent need to embed these dimensions into personalized prevention, precision diagnostics, and tailored therapeutic strategies to reduce inequities in breast cancer outcomes.

## Introduction

Breast cancer remains a major global health challenge, not only because of its high incidence and mortality but also due to persistent disparities in diagnosis, prognosis, and survival across demographic groups. These disparities are especially pronounced by age and race or ethnicity, underscoring a critical need for precision approaches in breast cancer research and care^1,2^. Mounting evidence indicates that outcome gaps stem from a complex interplay of tumor biology, genetic predisposition, access to care, and treatment responsiveness—factors that are further influenced by demographic and sociocultural determinants^3–7^.

The incidence of breast cancer in young women is increasing at an alarming rate^8^. Compared to older patients, young women diagnosed with breast cancer experience disproportionate psychosocial and clinical burdens. Early-age onset interrupts fertility, diminishes quality of life, impairs body image, and derails long-term life planning, compounding the physical toll of the disease^9,10^. Breast cancers in young women disproportionately manifest as aggressive subtypes such as triple-negative breast cancer (TNBC) and HER2-positive disease, which carry poorer prognoses and limited targeted therapies^11–13^. In contrast, breast cancers in older women are predominantly hormone receptor-positive, offering better prognosis and broader treatment options^14^.

Racial and ethnic factors further stratify breast cancer biology and outcomes. Young Asian women frequently exhibit more aggressive tumor biology and differential treatment responses compared to older patients and non-Asian counterparts^15–17^. For example, HER2-positive breast cancer occurs in ~18.7% of Asian women compared to 13.8% in White women, a difference particularly pronounced among younger patients13. In contrast, Black women are disproportionately affected by TNBC, a subtype associated with inferior survival outcomes and limited targeted therapies14. These racial disparities are compounded by systemic inequities: Black and Asian women are more likely to experience delays in diagnosis, reduced access to optimal treatment and follow-up, and higher rates of adverse events^18–21^.

Beyond demographic patterns, the tumor microenvironment and germline-mediated immunoediting play critical roles in shaping breast cancer subtypes, metastatic potential, and therapeutic response^22^. Recent multi-omic profiling has revealed patient-specific vulnerabilities, particularly among the Chinese population, enabling more refined patient stratification and personalized therapeutic targeting^23^. However, the Asian population is not monolithic; it encompasses heterogeneous subgroups with distinct molecular and clinical features, necessitating careful disaggregation in research.

Despite these advances, comprehensive studies integrating molecular subtypes, racial diversity, and menopausal stages remain limited—particularly in clarifying their combined effects on breast cancer incidence and survival disparities. Hormonal transitions across menopausal stages—premenopausal (<50 years), perimenopausal (50–64 years), and postmenopausal (≥65 years)—are thought to modulate tumor biology, yet these effects in racially diverse populations remain poorly characterized. In this study, we characterize breast cancer incidence and survival across age groups that broadly correspond to these hormonal stages, providing a framework to examine their combined impact with racial and subtype-specific factors.

To address these gaps, we analyzed Surveillance, Epidemiology, and End Results (SEER) datasets across two complementary time frames. The first dataset (1975–2022) was used to examine long-term trends in age-related breast cancer incidence and survival disparities, focusing on evolving hazard ratios between young (<50) and older (≥50) patients. The second dataset (2010–2022), which includes molecular subtype annotation, enabled investigation of contemporary patterns in incidence and survival jointly across age, race, and molecular subtype. Our aim is to leverage long-term trend data to characterize historical shifts and recent molecular subtype data to delineate contemporary patterns.

## Results

### Characteristics of the study population

The SEER 8 Registries dataset included 668,394 female breast cancer cases, of which 148,106 were classified as young (<50 years) and 520,288 as older (≥50 years). The SEER 17 Registries dataset comprised 718,365 female breast cancer cases, including 143,156 young cases (<50 years) and 575,209 older cases (≥50 years).

Within the SEER 17 dataset, the distribution of molecular subtypes was as follows: HR+/HER2− (Luminal A) constituted 74.21% (*n* = 533,112) of cases, HR+/HER2+ (Luminal B) 10.46% (*n* = 75,122), HR−/HER2+ (HER2-enriched) 4.43% (*n* = 31,854), and HR−/HER2− (Triple Negative) 10.90% (*n* = 78,277). Patients were categorized into five primary racial groups: non-Hispanic White (65.88%, *n* = 473,265), non-Hispanic Black (10.46%, *n* = 75,148), non-Hispanic Asian/Pacific Islander (9.77%, *n* = 70,196), Hispanic (12.80%, *n* = 91,946) and Other (1.09%, *n* = 7810). The non-Hispanic Asian/Pacific Islander group was further subdivided into specific ethnicities: Filipino (25.76%, *n* = 18,083), Chinese (20.91%, *n* = 14,675), Asian Indian or Pakistani (11.87%, *n* = 8333), Japanese (11.24%, *n* = 7892), Other Asian American (7.71%, *n* = 5,409), Korean (7.15%, *n* = 5020), Vietnamese (6.52%, *n* = 4577), Kampuchean (0.66%, *n* = 465), and Laotian (0.33%, *n* = 235), besides Native Hawaiian other Pacific Islander (7.85%, 5507). All reported patient numbers reflect the final cohort after excluding cases with missing HR or HER2 information. Detailed distributions of molecular subtypes across racial/ethnic and age groups are presented in Supplementary Table 1 and Supplementary Fig. 1.

### Incidence rate disparities by age, race/ethnicity, and molecular subtype

**Fig. 1:**
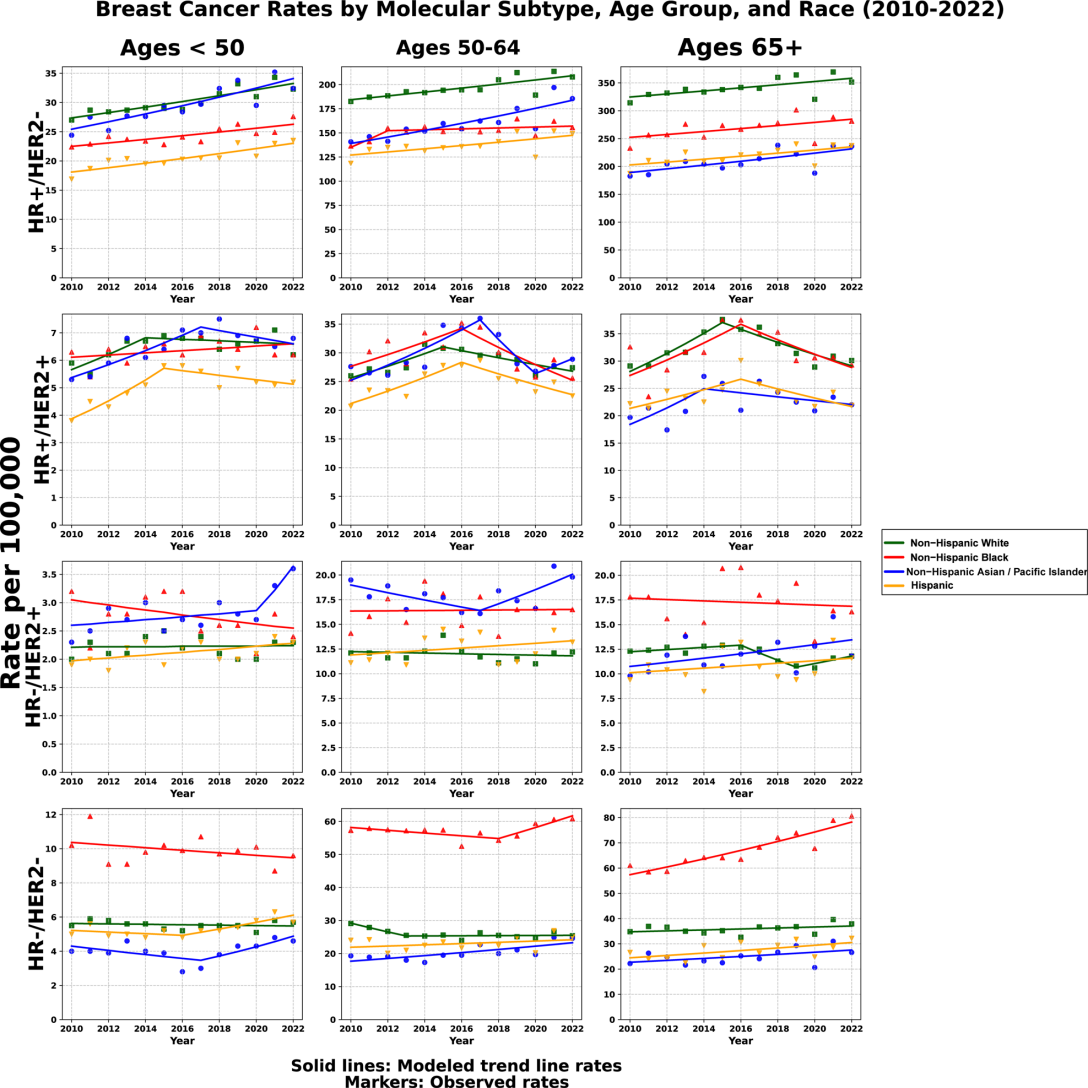
Breast cancer incidence rates from 2010 to 2022 by age group, molecular subtype, race/ethnicity, based on SEER incidence data.

We first examined age-adjusted breast cancer incidence rates (per 100,000 women) across subgroups defined by the intersection of age group, molecular subtype, and racial/ethnicity (Fig. 1).

For the HR+/HER2− subtype, non-Hispanic White women had the highest incidence rates across all age groups, although the magnitude of disparity varies by age when compared to other racial groups. Among women aged <50 years, incidence rates among non-Hispanic Asian/Pacific Islander women were similar to those observed in non-Hispanic White women. Differences between racial/ethnic groups were more pronounced in older age groups.

For the HR+/HER2+ subtype, incidence rates were comparable across racial/ethnic groups among women aged <50 and 50–64 years. In contrast, among women aged ≥65 years, non-Hispanic White and non-Hispanic Black women had higher incidence rates compared with other groups.

For the HR−/HER2+ subtype, incidence patterns varied by age. Among women aged <50 and 50–64 years, non-Hispanic Asian/Pacific Islander women had the highest incidence rates between 2010 and 2022. Among women aged ≥65 years, non-Hispanic Black women exhibited the highest rates. Joinpoint regression analyses indicated an increase in incidence rates among non-Hispanic Asian/Pacific Islander women after 2017 in the 50–64 age groups (annual percent change [APC], 4.11%; *p* < 0.0001). Similar patterns were observed in SEER Explorer data (Supplementary Fig. 2).

For the HR−/HER2− subtype, non-Hispanic Black women consistently exhibited the highest incidence rates across all age groups during the study period. Incidence rates in this group were approximately twice those observed among non-Hispanic White and non-Hispanic Asian/Pacific Islander women.

Age-specific distributions of molecular subtypes are presented in Supplementary Table 1. The proportion of HR + /HER2− tumors increased with age across all racial/ethnic groups, whereas the proportion of HR+/HER2+ tumors decreased with age. For HR−/HER2+ tumors, age-related patterns differed by race/ethnicity, with higher proportions observed among non-Hispanic Asian/Pacific Islander women aged 50–64 years. Triple-negative tumors (HR−/HER2−) represented a higher proportion of cases among non-Hispanic Black women across all age groups.

### Increased incidence of breast cancer cases among Asian and hispanic racial groups

**Fig. 2:**
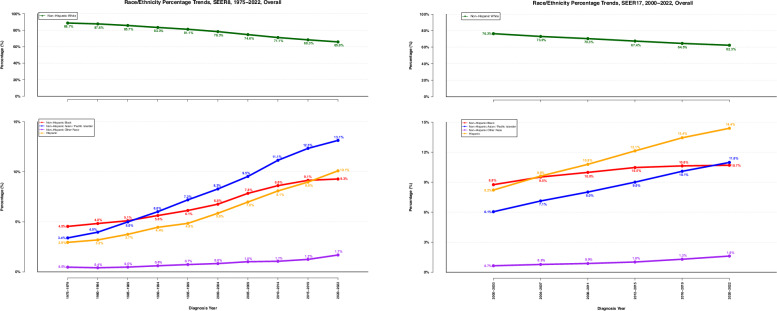
Temporal trends in the proportional distribution of breast cancer cases by race/ethnicity in the SEER 8 (1975–2022) and SEER 17 (2000–2022) registries. Non-Hispanic White (green), Non-Hispanic Black (red), Non-Hispanic Asian/Pacific Islander (blue), Hispanic (yellow), and Other (purple) patients are shown.

Figure 2 illustrate the shifting racial distribution of breast cancer cases in the SEER 8 and SEER 17 registries datasets, respectively. The data are grouped into five-year (SEER 8) and four-year (SEER 17) diagnosis intervals, with each point representing the percentage of total cases attributed to non-Hispanic White, non-Hispanic Black, non-Hispanic Asian/Pacific Islander, Hispanic, and Other racial groups, summing to 100% per interval. A notable upward trend was observed among non-Hispanic Asian/Pacific Islander (blue) and Hispanic (yellow) populations over time, with the rate of increase in both groups exceeding that of non-Hispanic Black individuals. In the SEER 8 registries, the number of non-Hispanic Asian/Pacific Islander patients surpassed that of non-Hispanic Black patients around 1990, and in the SEER 17 registries this crossover occurred around 2020. Despite differences in registry coverage, both datasets reveal a consistent pattern: the proportional representation of non-Hispanic Asian/Pacific Islander and Hispanic patients has increased steadily over time, surpassing that of non-Hispanic Black patients, whose growth has plateaued and even begun to decline. Supplementary Figs. 3, 4, which stratify patients by age group (young vs. older) for the SEER 8 and SEER 17 registries, further highlight this pattern, with the rise being particularly pronounced among young patients. Further analysis of Asian subgroups revealed notable shifts in proportional representation over time. The proportion of Japanese patients declined from 31.6% in 2000 to 15.8% in 2022, representing nearly a 50% relative decrease. In contrast, Chinese and Filipino patients showed steady increases and ranked as the first and second largest Asian subgroups in recent years (Supplementary Fig. 5). Survival curves for each Asian subgroup, stratified by age group and molecular subtype, are presented in Supplementary Fig. 6. Overall, most subtype-specific comparisons did not demonstrate statistically significant differences between Asian subgroups. The only significant differences were observed among patients aged 18–49 and 50–64 years with HR+/HER2− tumors. We note that limited sample sizes within specific subgroup–age–subtype intersections may have reduced statistical power to detect differences.

To provide additional context regarding stage at diagnosis, we examined temporal trends in SEER Combined Summary Stage (Localized, Regional, Distant), shown in Supplementary Fig. 7. Stage distributions were broadly comparable across racial/ethnic groups over time, with only modest differences observed, including a slightly higher proportion of distant-stage disease among Black patients.

### Temporal trends in age-related mortality disparities

**Fig. 3:**
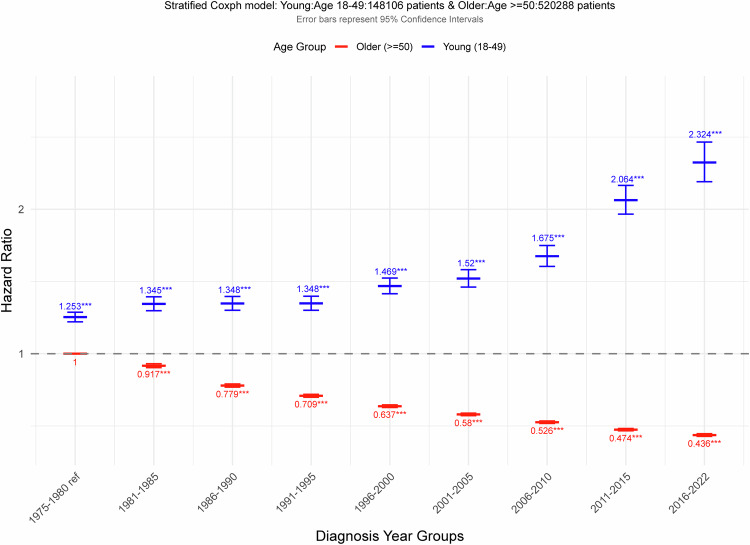
Diverging trends in mortality hazard ratios (HRs) for young (<50 years) and older (≥50 years) breast cancer patients, 1975–2022. Hazard ratios are presented with 95% confidence intervals. Asterisks indicate statistical significance for comparisons with the reference group (***p < 0.001).

In the SEER 8 registries dataset (1975–2022), we evaluated long-term mortality differences between young (<50 years) and older (≥50 years) patients using a Cox proportional hazards model with an interaction term between age group and diagnosis period (Fig. 3), adjusted for continuous age and other covariates. Using older patients diagnosed in 1975–1980 as the reference group, the hazard ratio for young patients increased from 1.253 (95% CI, 1.221–1.287) in 1975–1980 to 2.324 (95% CI, 2.191–2.465) in 2016–2022 (*p* < 0.0001 for interaction). In contrast, mortality hazards among older patients declined over time relative to the earliest diagnosis period (HR 0.436, 95% CI, 0.428–0.446 in 2016–2022).

### Survival disparities by age, race/ethnicity, and molecular subtype

In intra-molecular analyses (Table 1), hazard ratios (HRs) of mortality were estimated across racial/ethnic and age groups within each molecular subtype, using young (18–49 years) non-Hispanic White patients within each subtype as the reference group.

**Table 1:** Intra-molecular comparisons of hazard ratios for mortality across age and racial/ethnic groups

Molecular subtype	Racial group	HR (95% CI): 18–49	HR (95% CI): 50–64	HR (95% CI): ≥ 65
HR+/HER2−	non-Hispanic White	1 (Ref)	0.54 (0.51–0.58)^***^	0.41 (0.37–0.45)^***^
	non-Hispanic Black	2.33 (2.12–2.56)^***^	1.11(1.02–1.21)^**^	0.63 (0.56–0.70)^***^
	non-Hispanic Asian or Pacific Islander	0.92 (0.82–1.03)	0.51(0.45–0.56)^***^	0.33 (0.29–0.37)^***^
	Hispanic (All races)	1.46 (1.33–1.60)^***^	0.70 (0.64–0.77)^**^	0.43 (0.38–0.48)^***^
	Other	1.15 (0.81–1.62)	0.54 (0.41–0.70) ^***^	0.39 (0.30–0.49)^***^
HR+/HER2+	non-Hispanic White	1 (Ref)	0.67 (0.58–0.78)^***^	0.55 (0.45–0.68)^***^
	non-Hispanic Black	1.99 (1.64–2.41)^***^	1.17 (0.97–1.40)	0.75 (0.59–0.96)^*^
	non-Hispanic Asian or Pacific Islander	0.89 (0.70–1.14)	0.56 (0.45–0.70)^***^	0.46 (0.35–0.61)^***^
	Hispanic (All races)	1.26 (1.04–1.53)^**^	0.80 (0.66–0.97)^*^	0.62 (0.48–0.80)^***^
	Other	1.30 (0.69–2.43)	0.70 (0.41–1.19)	0.23 (0.10–0.56)^***^
HR−/HER2+	non-Hispanic White	1 (Ref)	0.66 (0.53–0.80)^***^	0.56 (0.42–0.75)^***^
	non-Hispanic Black	1.87 (1.46–2.40)^***^	1.03 (0.81–1.30)	0.76 (0.55–1.04)
	non-Hispanic Asian or Pacific Islander	0.81 (0.59–1.12)	0.59 (0.45–0.77)^***^	0.40 (0.28–0.58)^***^
	Hispanic (All races)	1.40 (1.10–1.78)^**^	0.73 (0.57–0.93)^**^	0.61(0.43–0.85)^***^
	Other	1.24 (0.54–2.84)	0.63 (0.31–1.25)	0.69 (0.34–1.39)
HR−/HER2−	non-Hispanic White	1 (Ref)	0.62 (0.56–0.69)^***^	0.52(0.44–0.60)^***^
	non-Hispanic Black	1.49 (1.33–1.68)^***^	0.90 (0.80–1.01)	0.63 (0.53–0.74)^***^
	non-Hispanic Asian or Pacific Islander	0.93 (0.77–1.13)	0.52 (0.43–0.63)^***^	0.42 (0.34–0.52)^***^
	Hispanic (All races)	1.25 (1.11–1.41)^***^	0.72 (0.62–0.83)^***^	0.56 (0.46–0.67)^***^
	Other	0.76 (0.44–1.32)	0.72 (0.47–1.10)	0.44 (0.27–0.70)^***^

This table presents hazard ratios (HRs) for overall mortality across racial/ethnic and age groups within each molecular subtype. Within each subtype, young non-Hispanic White patients served as the reference group. HR estimates were derived from Cox proportional hazards models including main effects for age group and race/ethnicity, as well as their interaction, and were adjusted for continuous age and other covariates. Hazard ratios are presented with 95% confidence intervals (CIs).

Asterisks indicate statistical significance: *p < 0.05; **p < 0.01; ***p < 0.001.

Across all molecular subtypes, mortality risk declined with increasing age. Women aged 50–64 and ≥65 years consistently exhibited lower HRs compared with those aged 18–49, with the most pronounced age-associated reduction observed in HR + /HER2− tumors.

Racial disparities were most evident among younger women (<50 years). In this age group, non-Hispanic Black women consistently exhibited the highest mortality across all molecular subtypes, whereas Hispanic women demonstrated moderately elevated risks. The magnitude of excess risk among Black women attenuated with advancing age, and disparities narrowed substantially in older age groups.

Racial differences were particularly apparent in hormone receptor–positive and HER2-positive subtypes. In triple-negative disease (HR−/HER2−), although Black women remained at elevated risk in younger patients, the relative differences were less pronounced, reflecting the uniformly aggressive biology of this subtype. Across all subtypes and age strata, Asian women consistently exhibited the lowest HRs, representing the most favorable survival profile. Supplementary Fig. 8 illustrates these survival patterns.

In the intra-racial analysis (Table 2), an age effect was evident across all racial groups, with hazard ratios generally declining with increasing age. The steepest age-related reductions were observed among non-Hispanic White patients, whereas Black patients showed comparatively smaller declines across subtypes.

**Table 2:** Intra-racial comparisons of hazard ratios for mortality across molecular subtypes and age groups

Racial Group	Molecular subtype	HR (95% CI): 18–49	HR (95% CI): 50–64	HR (95% CI): ≥ 65
Non-Hispanic White	HR+/HER2−	1 (ref)	0.50 (0.47–0.53)^***^	0.34 (0.31–0.37)^***^
	HR+/HER2+	1.11(0.99–1.24)	0.74 (0.67–0.81)^***^	0.59 (0.54–0.66)^***^
	HR−/HER2+	1.73 (1.49–2.01) ^***^	1.05 (0.94–1.16)	0.84 (0.75–0.95)^***^
	HR−/HER2−	3.11 (2.86–3.38)^***^	1.50 (1.39–1.62)^***^	0.99 (0.90–1.09)
Non-Hispanic Black	HR+/HER2−	1 (ref)	0.67 (0.59–0.75)^***^	0.54 (0.45–0.64)^***^
	HR+/HER2+	0.92 (0.78–1.10)	0.82 (0.70–0.97)^**^	0.82 (0.66–1.01)
	HR−/HER2+	1.40 (1.13–1.73)^***^	1.08 (0.89–1.29)	1.20 (0.95–1.52)
	HR−/HER2−	2.02 (1.79–2.27)^***^	1.46 (1.29–1.65)^***^	1.28 (1.07–1.54)^**^
Non-Hispanic Asian or Pacific Islander	HR+/HER2−	1 (ref)	0.60 (0.51–0.71)^***^	0.42 (0.33–0.55)^***^
	HR+/HER2+	1.07 (0.84–1.36)	0.78 (0.62–0.98)^*^	0.77 (0.57–1.04)
	HR−/HER2+	1.56 (1.16–2.11)^***^	1.22 (0.97–1.55)	0.95 (0.68–1.34)
	HR−/HER2-	3.24 (2.65–3.96)^***^	1.67 (1.35–2.07)^***^	1.28 (0.96–1.71)
Hispanic (All races)	HR+/HER2−	1 (ref)	0.61 (0.54–0.70)^***^	0.50 (0.41–0.61)^***^
	HR+/HER2+	0.95 (0.80–1.13)	0.83 (0.70–0.98)^*^	0.93 (0.74–1.18)
	HR−/HER2+	1.70 (1.39–2.08)^***^	1.13 (0.93–1.38)	1.33 (1.02–1.74)^*^
	HR−/HER2−	2.77 (2.45–3.12)^***^	1.75 (1.51–2.02)^***^	1.58 (1.27–1.96)^***^
Other	HR+/HER2−	1 (ref)	0.55 (0.33–0.92)^*^	0.46 (0.22–0.98)^*^
	HR+/HER2+	1.24 (0.62–2.48)	0.85 (0.43–1.65)	0.37 (0.12–1.11)
	HR−/HER2+	1.89 (0.80–4.51)	1.12 (0.51–2.46)	1.51 (0.58–3.92)
	HR−/HER2−	2.08 (1.11–3.91)^*^	1.98 (1.09–3.62)^*^	1.28 (0.55–2.95)

This table presents hazard ratios (HRs) for overall mortality across molecular subtype and age groups within each racial/ethnicity. Within racial group, patients with HR+/HER2– (Luminal A) tumors aged 18–49 years serve as the reference group. HR estimates were derived from Cox proportional hazards models including main effects for age group and molecular subtype, as well as their interaction, and were adjusted for continuous age and other covariates. Hazard ratios are presented with 95% confidence intervals (CIs).

Asterisks indicate statistical significance: *p < 0.05; **p < 0.01; ***p < 0.001.

By molecular subtype, the most pronounced age-related reduction occurred in HR+/HER2− tumors across all racial groups. In contrast, both HER2-positive subtypes (HR+/HER2+ and HR−/HER2+) demonstrated more modest age-related declines, with hazard ratios remaining comparatively elevated in older age groups relative to HR+/HER2− disease.

Across all racial groups, young women with triple-negative disease (HR−/HER2−) exhibited the highest mortality risks compared with other subtypes. Among young patients, triple-negative tumors were associated with hazard ratios of 3.24 in Asian women and 3.11 in White women, whereas Black women showed a lower—though still markedly elevated—risk (HR 2.02), indicating comparatively less extreme subtype contrasts. In the 50–64 age group, triple-negative tumors continued to confer substantial excess risk across racial categories, although the magnitude was attenuated relative to younger patients.

Notably, Black patients displayed smaller variation in hazard ratios across molecular subtypes and age strata compared with other racial groups, suggesting attenuated subtype-related heterogeneity within this population.

Survival curves (Supplementary Fig. 9), stratified by race, age group, and molecular subtype, visually corroborate these patterns.

Taken together, results from both intra-molecular and intra-racial analyses revealed consistent patterns. Age effects were evident across all races and molecular subtypes, with progressively lower hazard ratios in older age groups. The age-associated decline was most pronounced in HR+/HER2− tumors and less evident in HER2-positive subtypes. White patients demonstrated the steepest reductions with age, whereas Black patients exhibited persistently elevated risks and comparatively weaker age gradients.

Survival disparities were greatest in younger women—both across racial groups and across molecular subtypes—and diminished with advancing age. Among subtypes, triple-negative (HR−/HER2−) disease consistently conferred the highest relative risk. Although Asian patients exhibited relatively high hazard ratios in certain subtype–age strata, Black patients showed the least variation across subtypes and age groups, indicating reduced subtype-driven heterogeneity relative to other racial groups.

## Discussion

Our retrospective analysis of nearly 700,000 women with breast cancer over five decades demonstrates the complex and intersecting effects of age, race/ethnicity, and molecular subtype on incidence and mortality in the United States. Survival disparities were most pronounced among younger patients (18–49 years), whose mortality risks have not improved to the same extent as those of older women. At the same time, incidence rates have risen among Asian and Hispanic populations, with particularly notable increases in HER2-positive disease among Asian women. Although Asian women overall exhibited lower mortality, age- and subtype-specific analyses revealed high-risk subgroups—especially younger women with triple-negative disease. Collectively, these findings underscore the importance of examining breast cancer outcomes through an intersectional framework that integrates age, race/ethnicity, and tumor biology.

Our findings align with recent SEER-based incidence analyses, which documented increasing breast cancer incidence among non-Hispanic Asian or Pacific Islander and Hispanic women, particularly in younger age groups^24^. Similarly, *Breast Cancer Statistics 2024* reported that Asian/Pacific Islander (AAPI) women experienced the fastest rising incidence rates in the United States, especially among younger women^25^. Our analysis extends these observations by identifying the specific molecular subtypes contributing to this rise. While prior work has highlighted subtype-specific mortality disparities within Asian populations^26^, few studies have examined these patterns among U.S. Asian populations across age and molecular subtype strata. Importantly, relatively few population-based analyses have simultaneously evaluated age-, subtype-, and race/ethnicity-specific mortality patterns in an intersectional framework^27^. Comparable comprehensive evaluations in the U.S. population—particularly those incorporating disaggregated Asian subgroups—remain limited. Our study, therefore, provides a more granular understanding of how demographic and biological factors jointly shape breast cancer disparities in the United States.

Leveraging nearly five decades of data, we further demonstrate that the survival gap between younger and older women has widened over time. Although survival improved substantially among older patients, younger women did not experience comparable gains; in relative terms, the mortality hazard ratio comparing younger to older women more than doubled since 1975. This divergence challenges the narrative of uniformly improving breast cancer survival and highlights the disproportionate burden borne by younger women, who face greater years of potential life lost.

We also observed a shift in the racial/ethnic composition of breast cancer cases, with the proportion of Asian patients surpassing that of Black patients in recent years within SEER registries. Although this pattern reflects broader U.S. demographic changes—including growth in Hispanic and Asian populations—the absolute increase in Asian cases, particularly in specific molecular subtypes, suggests evolving epidemiologic dynamics beyond population structure alone.

Black women, consistent with prior reports, bore the highest mortality risk across nearly all subtypes. Yet, our results reveal a subtler nuance: Black patients exhibited less variation in hazard ratios across subtypes compared with other racial groups, suggesting the importance of moving beyond broad racial categories to examine intersectional patterns of age, race, and molecular subtype.

Several mechanisms may contribute to these disparities, although they cannot be directly assessed in SEER. Differences in tumor biology, treatment access and patterns, and structural inequities in screening and survivorship care may all play roles. The higher prevalence of TNBC among Black women, combined with documented inequities in access to high-quality care, likely contributes to persistent survival disadvantages. Similarly, variability in treatment access or response may influence outcomes within specific Asian subgroups.

These findings have important clinical and research implications. They highlight the need for age-stratified and subtype-specific approaches to risk assessment, particularly for high-risk groups such as young Black women with TNBC and younger or middle-aged Asian women with HER2-positive disease. They also underscore the importance of integrative research examining how biological and structural factors intersect across the cancer continuum, as well as improved representation of Asian and Hispanic populations in clinical and population-based studies.

This study has several limitations. SEER lacks detailed information on systemic therapies, including chemotherapy, endocrine therapy, HER2-targeted agents, and immunotherapy, limiting evaluation of treatment-related contributions to survival disparities. Individual-level socioeconomic variables are also unavailable. Molecular subtype data are only available beginning in 2010, restricting long-term subtype-specific analyses. Although Asian patients were analyzed as a group, substantial heterogeneity exists within Asian subpopulations that may not be fully captured. Additionally, while stage at diagnosis is an important prognostic factor, stage-specific survival and incidence analyses were not the primary focus of this study. Finally, stage-specific incidence rates were not computed across all intersectional strata due to high-dimensional stratification and sparse subgroup counts.

Overall, our findings reveal an emerging demographic and molecular shift in breast cancer epidemiology, marked by disproportionate burdens among younger women, Asian and Hispanic populations, and biologically aggressive subtypes. Integrating age, race/ethnicity, and molecular subtype into research design and clinical practice may advance precision prevention, diagnosis, and treatment strategies that more effectively address disparities, reduce inequities, and improve survival outcomes across diverse populations.

## Methods

### Patient collection

We analyzed data from the SEER 8 Registries (1975–2022) to evaluate long-term trends in young breast cancer patients. This database represents approximately 8.3% of the U.S. population. We also used data from the SEER 17 Registries (2000–2022), which provides detailed molecular subtype information and covers ~26.5% of the U.S. population based on the 2020 census, for subtype-specific cross-sectional analyses.

All female breast cancer patients were identified using the ICD-O-3/WHO 2008 Site Recode criteria (Supplementary Fig. 10). Because detailed molecular subtype information has only been available for cases diagnosed after 2010, subtype-specific analyses were restricted to this period. Patients younger than 18 years were excluded due to the rarity and distinct biology of pediatric breast cancer. For patients with duplicate entries, only the first occurrence was retained. Extracted clinical and demographic variables included age at diagnosis, race/ethnicity, marital status, year of diagnosis, tumor stage, hormone receptor (HR) and HER2 status, histological subtype, and follow-up status (alive or deceased, with overall survival [OS] time). After applying these inclusion and exclusion criteria, we defined two analytic cohorts: one for longitudinal trend analyses and another for subgroup-specific analyses.

Breast carcinomas were categorized into four molecular subtypes based on HR and HER2 status, following established immunohistochemistry-based surrogate definitions^28^: HR+/HER2− (Luminal A), HR+/HER2+ (Luminal B), HR−/HER2+ (HER2-enriched), and HR−/HER2− (triple-negative). Patients diagnosed before age 50 were classified as early-onset (young), whereas those diagnosed at age 50 or older were considered older patients^29^.

Race and ethnicity were defined using SEER classifications, grouped into the following mutually exclusive categories: non-Hispanic White, non-Hispanic Black, non-Hispanic Asian/Pacific Islander, Hispanic, and non-Hispanic Other. The Asian component of non-Hispanic Asian/Pacific Islander included subgroups such as Filipino, Chinese, Japanese, Asian Indian or Pakistani, Korean, Vietnamese, Laotian, Kampuchean, and other Asian ethnicities, collectively referred to as “Other Asian”. Native Hawaiian and Pacific Islander individuals were included in the same group. Patients not fitting into these categories were classified as “non-Hispanic Other”.

### Statistical analysis

To investigate temporal changes in age-related survival disparities, we fitted a two-way interaction Cox proportional hazards (CoxPH) model between year of diagnosis and age group (young <50 vs. older ≥50) using the SEER 8 Registries dataset. This model estimated long-term trends in the relative hazard ratio (HR) for young versus older patients. Continuous age was included as a covariate to capture the overall effect of aging on survival, while categorical age allowed assessment of whether a young age at diagnosis conferred an additional survival disadvantage. A significant effect of the young age group, after adjusting for continuous age, would suggest that early-onset breast cancer represents a distinct clinical or biological entity with intrinsically poorer outcomes.

To comprehensively assess survival disparities across molecular subtypes and racial/ethnic groups, we further applied stratified CoxPH models using the SEER 17 Registries dataset (2010–2022). First, an intra-molecular analysis was performed: patients were stratified by molecular subtype, and within each subtype, hazard ratios (HRs) were estimated across racial/ethnic and age groups, using young White patients as the reference. Second, an intra-racial analysis was conducted: patients were stratified by racial/ethnic group, and within each group, HRs were estimated across molecular subtypes and age groups, using young patients with HR+/HER2– (Luminal A) as the internal reference subtype. Age was categorized into three groups (0–49, 50–64, ≥65 years), with the latter two reflecting physiological differences around menopausal status. Continuous age was also included to adjust for underlying aging effects. The proportional hazards assumption was considered valid, and no time-varying interactions were specified.

To explore potential heterogeneity in incidence rates, we used the SEER 17 Registries dataset to perform subgroup analyses and computed incidence rates across racial/ethnicity, molecular subtypes, and age groups, with rates age-adjusted to the US standard population. Individual cancer records were retrieved, and adjusted incidence rates generated using SEER*Stat version 9.0.41. To examine temporal patterns from 2010 to 2022, we fitted Joinpoint regressions to the natural log–transformed annual adjusted incidence rates. Trend analyses were performed with the NCI Joinpoint regression software version 5.40 (National Cancer Institute).

All statistical tests were two-sided, with *p*-values < 0.05 considered statistically significant. Analyses were conducted in R (version 4.3.2) using the survival (version 3.7-0) and survminer (version 0.4.9) packages.

### Ethics statement

This study used publicly available, de-identified data from the Surveillance, Epidemiology, and End Results (SEER) Program. Therefore, institutional review board approval and informed consent were not required under Houston Methodist Hospital policies. All procedures complied with the ethical standards of the Declaration of Helsinki (2013 revision).

## Supplementary information

41523_2026_935_MOESM1_ESM

## Data Availability

The data analyzed in this study were obtained from the Surveillance, Epidemiology, and End Results (SEER) Program. These data are publicly available and can be accessed through the SEER*Stat software upon request and registration with the SEER Program.

## Code availability

All code used for the data analysis and visualization in this study is publicly available at https://github.com/methodistsmab/young-cancer.
