# Supplementary material for: Disparities in breast cancer incidence and survival by age, race, and molecular subtype in US women
Source: NPJ Breast Cancer. 2026 Mar 27;12:97. doi: 10.1038/s41523-026-00935-y (PMC13415841; doi:10.1038/s41523-026-00935-y)
Supplement: Supplementary file 1 — 41523_2026_935_MOESM1_ESM [file 41523_2026_935_MOESM1_ESM.docx]

**Supplementary Table 1.** Distribution of Breast Cancer Molecular Subtypes by Race/Ethnicity and Age Group. Each row presents the count and percentage of cases, normalized within each racial/ethnic and age group, based on the SEER 17 Registries dataset (2010–2022).

**Supplementary Figure 1.** Distribution of Racial/Ethnic Groups and non-Hispanic Asian/Pacific Islander Subgroups. Percentages represent the proportion of each racial/ethnic group (left) and the proportion of each non-Hispanic Asian/Pacific Islander subgroup relative to the total non-Hispanic Asian/Pacific Islander population (right) in the SEER 17 Registries dataset.

**Supplementary Figure 2.** Breast cancer incidence rates by molecular subtype, age at diagnosis, and race/ethnicity, based on SEER*Explorer data from the most recent period (2018–2022), using the original data as presented in SEER**Explorer.*

**Supplementary Figure 3.** Temporal trends in the proportional distribution of breast cancer cases by race/ethnicity in the SEER 8 registries (1975–2022). Non-Hispanic White (green), non-Hispanic Black (red), non-Hispanic Asian/Pacific Islander (blue), Hispanic (yellow), and Other (purple) patients are shown separately for older (left) and younger (right) age groups.

**Supplementary Figure 4.** Temporal trends in the proportional distribution of breast cancer cases by race/ethnicity in the SEER 17 registries (2000–2022). Non-Hispanic White (green), non-Hispanic Black (red), non-Hispanic Asian/Pacific Islander (blue), Hispanic (yellow), and Other (purple) patients are shown separately for older (left) and younger (right) age groups.

**Supplementary Figure 5.** Temporal Trends in Asian Breast Cancer Subgroup Distribution in the SEER 17 Registries.

**Supplementary Figure 6.** Survival Analyses of Asian Subgroups Stratified by Molecular Subtype and Age Group. Kaplan–Meier survival curves are presented for major Asian subgroups across molecular subtypes and age groups (18–49, 50–64, and ≥65 years). Each panel displays subgroup-specific survival comparisons within a given age–subtype stratum. Most comparisons did not demonstrate statistically significant differences between Asian subgroups. Significant differences were observed only among patients aged 18–49 and 50–64 years with HR+/HER2− tumors. Limited sample sizes within specific subgroup–age–subtype intersections may have reduced statistical power.

**Supplementary Figure 7.** Temporal Trends in Breast Cancer Stage Distribution by Race/Ethnicity (SEER 17 Registries).

**Supplementary Figure 8.** Survival Analyses of Racial Groups Stratified by Molecular Subtypes and Age Groups. Kaplan–Meier survival curves are shown for each molecular subtype across age groups 0–49, 50–64, and ≥65 years. Each panel displays five survival curves representing the five racial groups, stratified by age and molecular subtype. Notably, hazard ratios for Black and Hispanic patients decreased with increasing age, indicating relatively better outcomes in older patients from these groups. In addition, survival disparities across racial groups were most pronounced among younger patients.

**Supplementary Figure 9.** Survival Analyses of Molecular Subtypes Stratified by Race/ethnicity and Age Groups. Kaplan–Meier survival curves are shown for each racial group across age groups 0–49, 50–64, and ≥65 years. Each panel displays four survival curves, representing the molecular subtypes, stratified by age and racial group. Notably, outcomes for HER2-positive groups worsened with increasing age, and their survival curves approached those of triple-negative breast cancer. This indicates that age-related reductions in hazard ratios were less pronounced for these two subtypes. In addition, among younger patients, survival disparities across molecular subtypes were the most pronounced.

**Supplementary Figure 10.** Flow Diagram for Study Cohort Inclusion.

**Supplementary Table 1.** Distribution of Breast Cancer Molecular Subtypes by Race/Ethnicity and Age Group. Each row presents the count and percentage of cases, normalized within each racial/ethnic and age group, based on the SEER 17 Registries dataset (2010–2022).

| Molecular subtype/  Race | HR+/HER2-  Luminal A | HR+/HER2+  Luminal B | HR-/HER2+  Enriched HER2 | HR-/HER2-  Triple Negative |
| --- | --- | --- | --- | --- |
| Total | **533112 (74.21%)** | **75122 (10.46%)** | **31854(4.43%)** | **78277 (10.90%)** |
| non-Hispanic White: 473,265 | **364700 (77.06%)** | **46333 (9.79%)** | **17686 (3.74%)** | **44546 (9.41%)** |
| non-Hispanic Black: 75148 | **47232 (62.85%)** | **8372 (11.14%)** | **4199 (5.59%)** | **15345 (20.42%)** |
| Asian/Pacific Islander: 70196 | **51045 (72.72%)** | **8419(11.99%)** | **4436 (6.32%%)** | **6296 (8.97%)** |
| Hispanic: 91946 | **64382 (70.02%)** | **11125 (12.10%)** | **5145 (5.60%)** | **11294 (12.28%)** |
| Other: 7810 | **5753 (73.66%)** | **873 (11.18%)** | **388 (4.97%)** | **796 (10.19%)** |
| non-Hispanic White: 18-49 | **51882 (68.20%)** | **11052 (14.53%)** | **3758 (4.94%)** | **9377 (12.33%)** |
| 50-64 | **125511 (75.32%)** | **17701 (10.62%)** | **7438 (4.46%)** | **15988 (9.59%)** |
| ≥65 | **187307 (81.24%)** | **17580 (7.62%)** | **6490 (2.81%)** | **19181 (8.32%)** |
| Black: 18-49 | **9887 (56.14%)** | **2616 (14.85%)** | **1116 (6.34%)** | **3993 (22.67%)** |
| 50-64 | **17905 (60.19%)** | **3447 (11.59%)** | **1872 (6.29%)** | **6522 (21.93%)** |
| ≥65 | **19440**  **(69.95%)** | **2309 (8.31%)** | **1211 (4.36%)** | **4830 (17.38%)** |
| Asian/Pacific Islander: 18-49 | **13475 (68.87%)** | **2982 (15.24%)** | **1291 (6.60%)** | **1818 (9.29%)** |
| 50-64 | **19113 (70.73%)** | **3444 (12.74%)** | **2107 (7.80%)** | **2359 (8.73%)** |
| ≥65 | **18457 (78.18%)** | **1993 (8.44%)** | **1038 (4.40%)** | **2119 (8.98%)** |
| Hispanic: 18-49 | **17500 (62.22%)** | **4267 (15.17%)** | **1811 (6.44%)** | **4549 (16.17%)** |
| 50-64 | **24825 (69.72%)** | **4472 (12.56%)** | **2261 (6.35%)** | **4051 (11.38%)** |
| ≥65 | **22057 (78.19%)** | **2386 (8.46%)** | **1073 (3.80%)** | **2694 (9.55%)** |
| Other: 18-49 | **1130 (63.41%)** | **290 (16.27%)** | **115 (6.45%)** | **247 (13.86%)** |
| 50-64 | **2296 (73.28%)** | **368 (11.75%)** | **180 (5.75%)** | **289 (9.22%)** |
| ≥65 | **2327 (80.38%)** | **215 (7.43%)** | **93 (3.21%)** | **260 (8.98%)** |


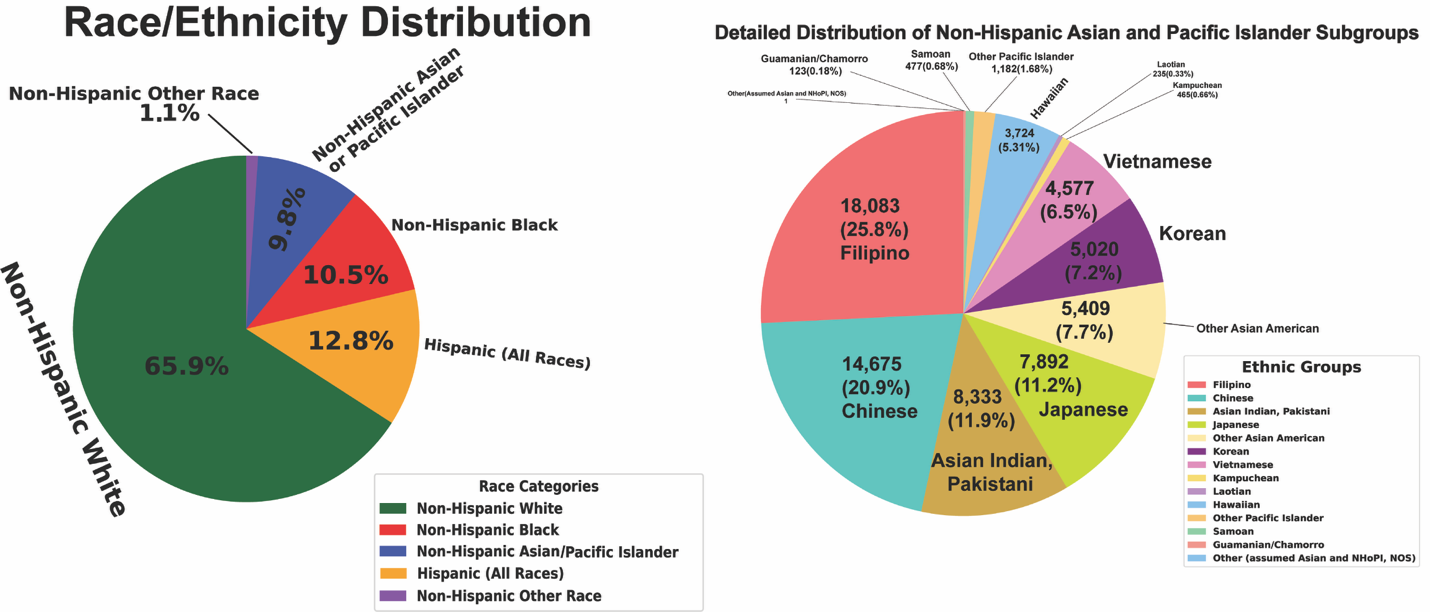


**Supplementary Figure 1.** Distribution of Racial/Ethnic Groups and non-Hispanic Asian/Pacific Islander Subgroups. Percentages represent the proportion of each racial/ethnic group (left) and the proportion of each non-Hispanic Asian/Pacific Islander subgroup relative to the total non-Hispanic Asian/Pacific Islander population (right) in the SEER 17 Registries dataset.


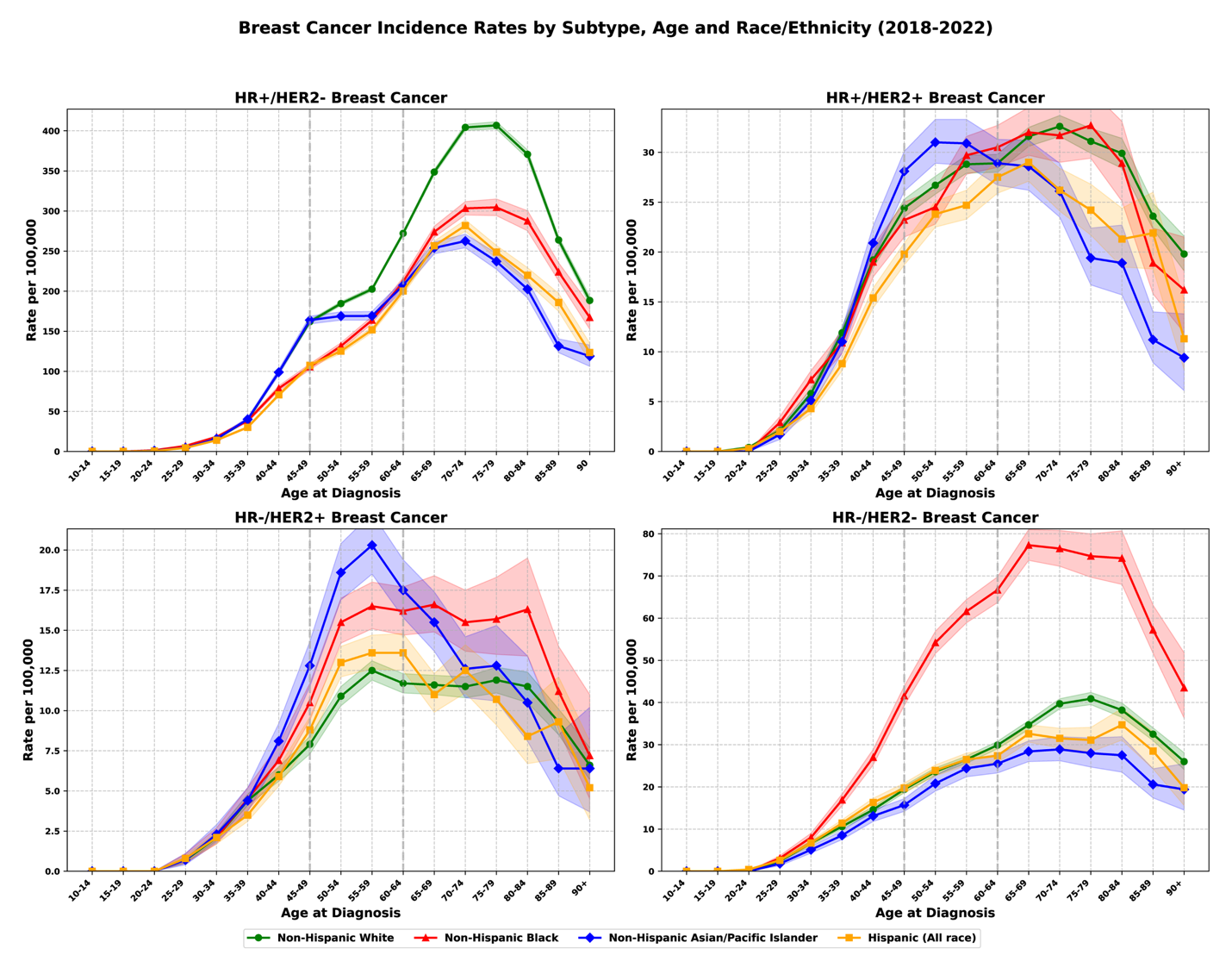


**Supplementary Figure 2.** Breast cancer incidence rates by molecular subtype, age at diagnosis, and race/ethnicity, based on SEER*Explorer data from the most recent period (2018–2022), using the original data as presented in SEER**Explorer.*

**Supplementary Figure 3.** Temporal trends in the proportional distribution of breast cancer cases by race/ethnicity in the SEER 8 registries (1975–2022). Non-Hispanic White (green), non-Hispanic Black (red), non-Hispanic Asian/Pacific Islander (blue), Hispanic (yellow), and Other (purple) patients are shown separately for older (left) and younger (right) age groups.

**Supplementary Figure 4.** Temporal trends in the proportional distribution of breast cancer cases by race/ethnicity in the SEER 17 registries (2000–2022). Non-Hispanic White (green), non-Hispanic Black (red), non-Hispanic Asian/Pacific Islander (blue), Hispanic (yellow), and Other (purple) patients are shown separately for older (left) and younger (right) age groups.


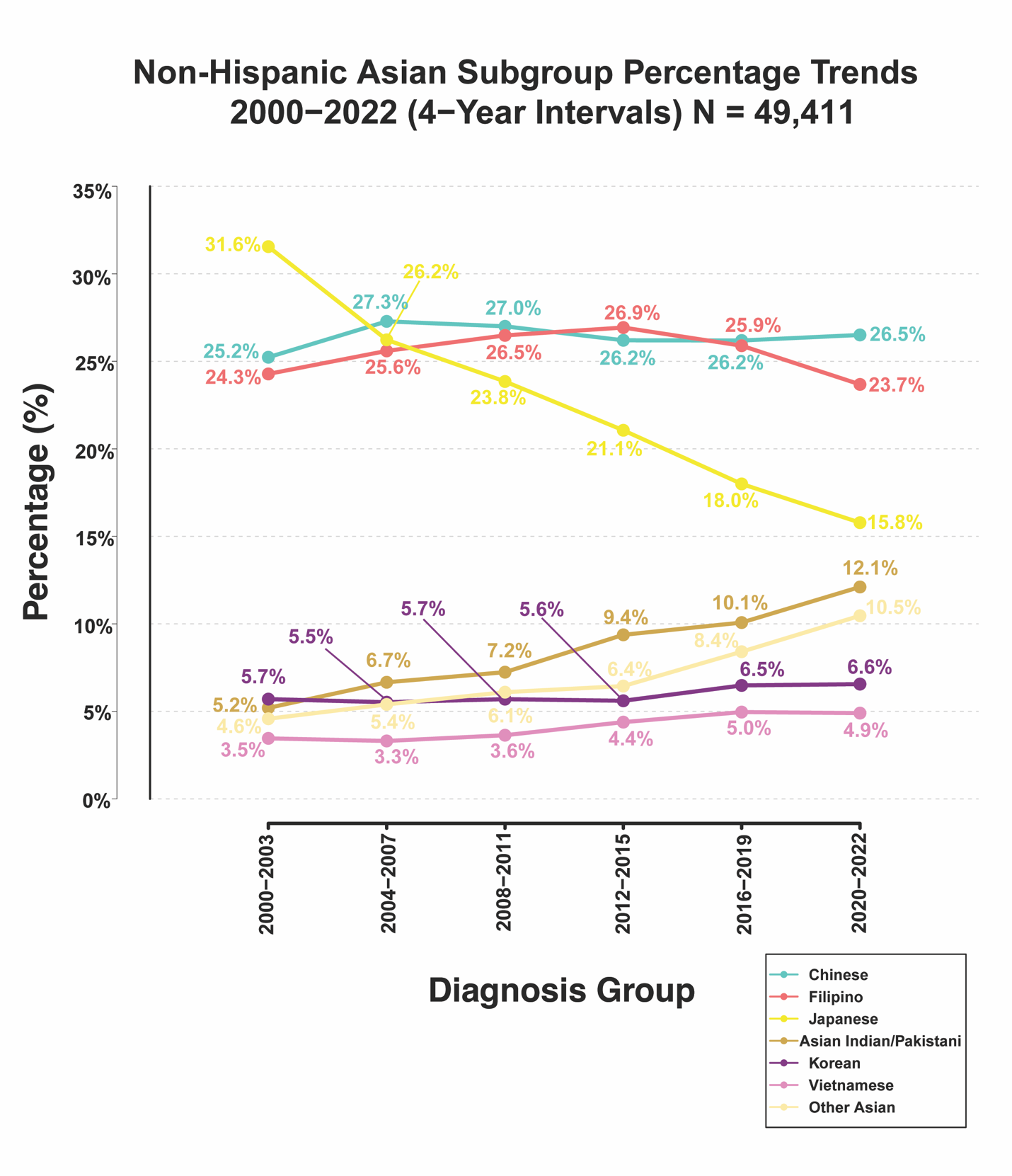


**Supplementary Figure 5.** Temporal Trends in Asian Breast Cancer Subgroup Distribution in the SEER 17 Registries.


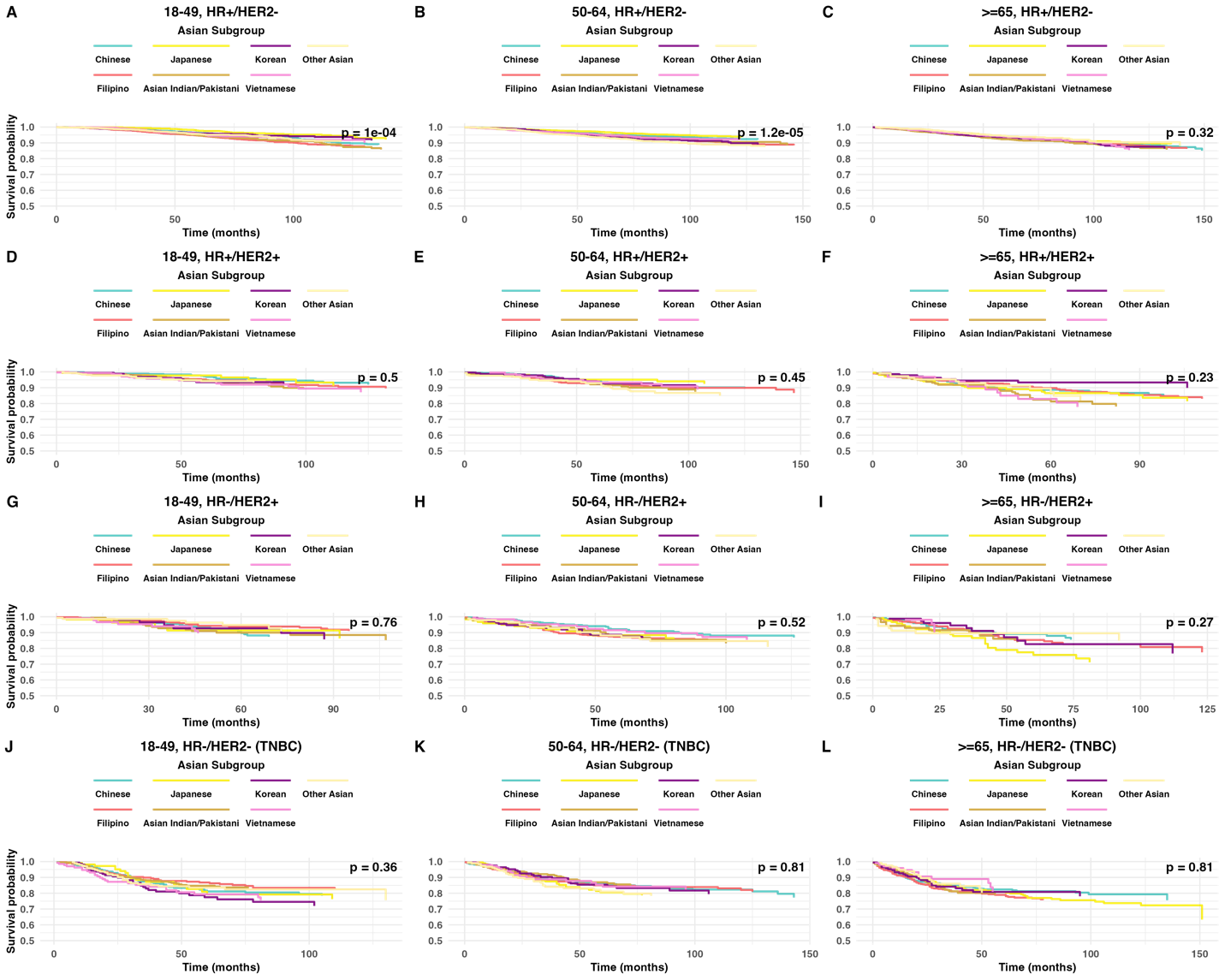


**Supplementary Figure 6.** Survival Analyses of Asian Subgroups Stratified by Molecular Subtype and Age Group. Kaplan–Meier survival curves are presented for major Asian subgroups across molecular subtypes and age groups (18–49, 50–64, and ≥65 years). Each panel displays subgroup-specific survival comparisons within a given age–subtype stratum. Most comparisons did not demonstrate statistically significant differences between Asian subgroups. Significant differences were observed only among patients aged 18–49 and 50–64 years with HR+/HER2− tumors. Limited sample sizes within specific subgroup–age–subtype intersections may have reduced statistical power.


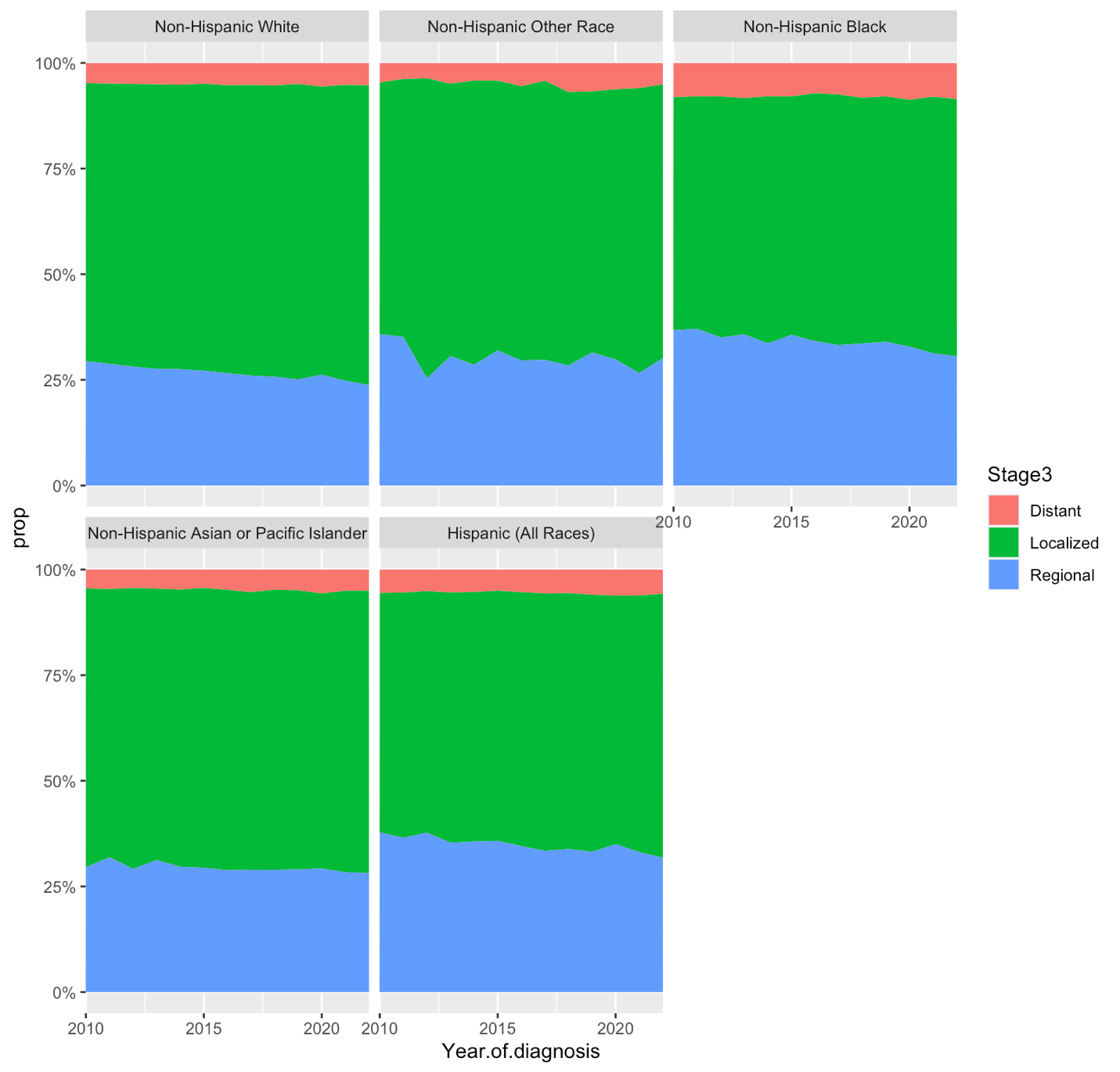


**Supplementary Figure 7.** Temporal Trends in Breast Cancer Stage Distribution by Race/Ethnicity (SEER 17 Registries).


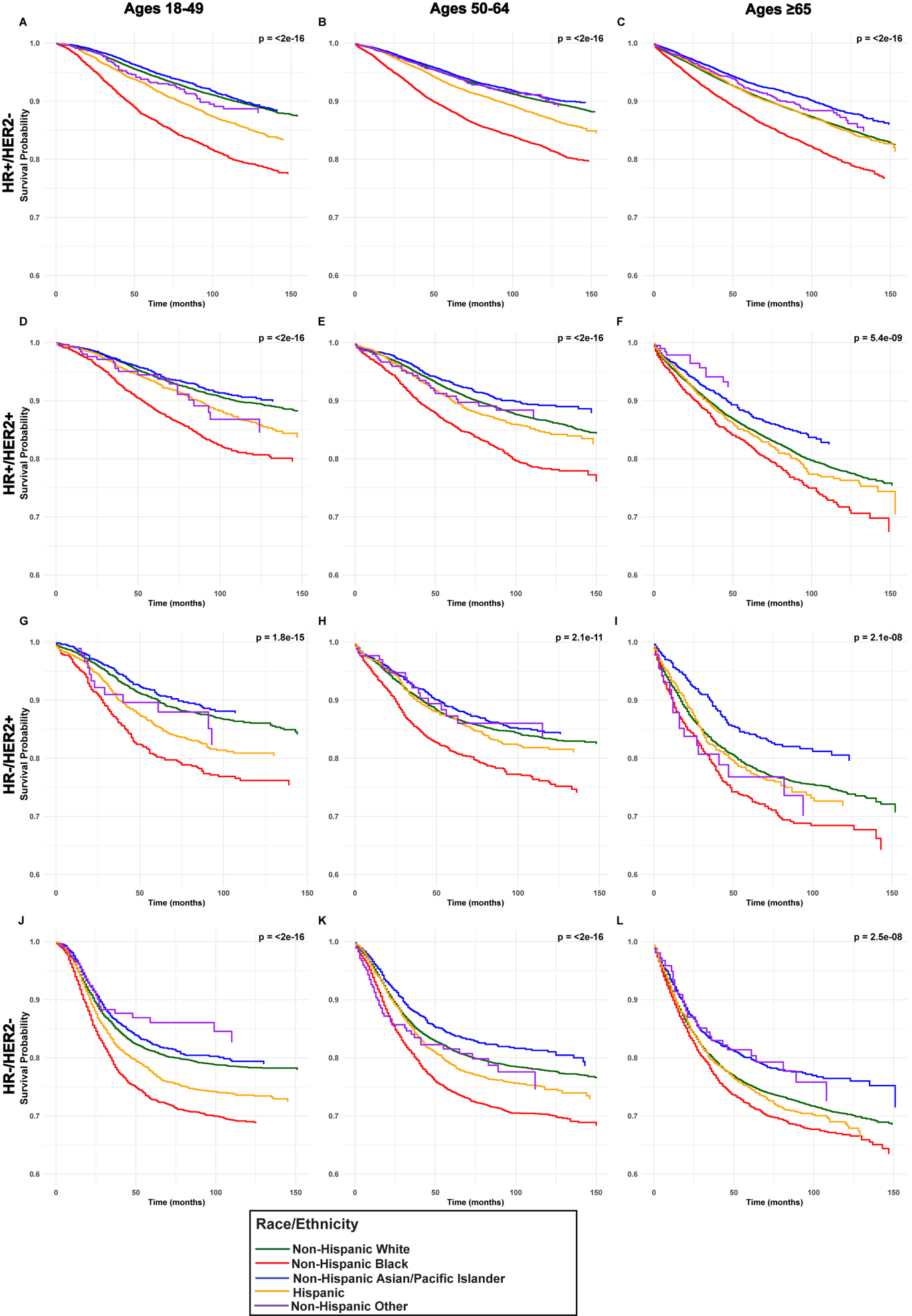


**Supplementary Figure 8.** Survival Analyses of Racial Groups Stratified by Molecular Subtypes and Age Groups. Kaplan–Meier survival curves are shown for each molecular subtype across age groups 0–49, 50–64, and ≥65 years. Each panel displays five survival curves representing the five racial groups, stratified by age and molecular subtype. Notably, hazard ratios for Black and Hispanic patients decreased with increasing age, indicating relatively better outcomes in older patients from these groups. In addition, survival disparities across racial groups were most pronounced among younger patients.


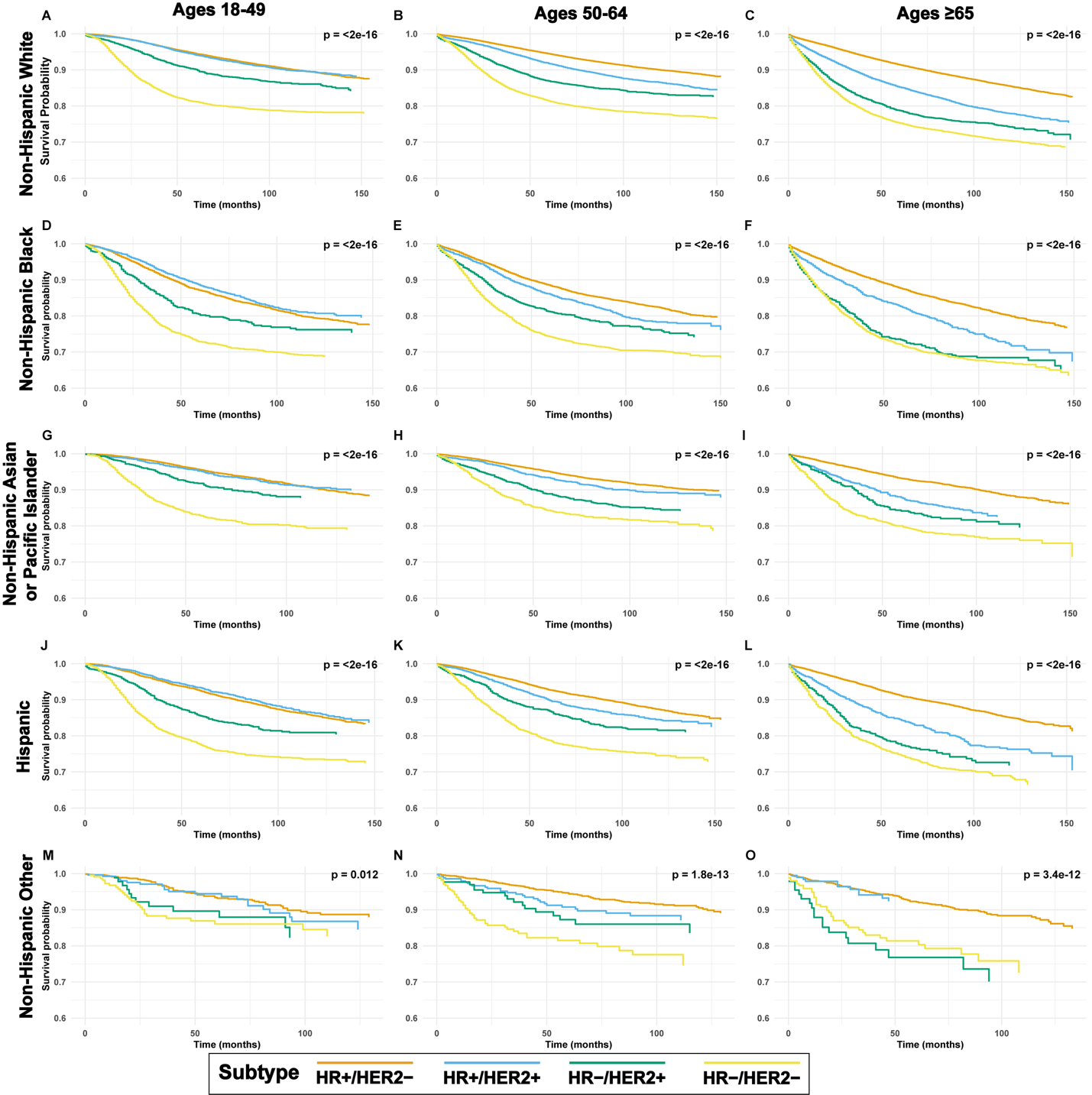


**Supplementary Figure 9.** Survival Analyses of Molecular Subtypes Stratified by Race/ethnicity and Age Groups. Kaplan–Meier survival curves are shown for each racial group across age groups 0–49, 50–64, and ≥65 years. Each panel displays four survival curves, representing the molecular subtypes, stratified by age and racial group. Notably, outcomes for HER2-positive groups worsened with increasing age, and their survival curves approached those of triple-negative breast cancer. This indicates that age-related reductions in hazard ratios were less pronounced for these two subtypes. In addition, among younger patients, survival disparities across molecular subtypes were the most pronounced.

**Supplementary Figure 10.** Flow Diagram for Study Cohort Inclusion.
